# Analysis of Clavien-Dindo classification and risk factors for complications after transurethral Thulium Laser enucleation of the prostate

**DOI:** 10.1097/MD.0000000000048446

**Published:** 2026-04-17

**Authors:** Chao Zuo, Wenzhi Gao, Yisen Meng, Kunlin Yang, Minghua Zhang, Xuebing Meng, Kai Zhang

**Affiliations:** a Department of Urology, Peking University First Hospital, Beijing, China; b Department of Urology, Peking University First Hospital - Miyun Hospital, Beijing, China.

**Keywords:** Benign Prostate Hyperplasia, Risk Factors, ThuLEP, Urethral Stricture

## Abstract

This study aimed to investigate the risk factors for postoperative complications following transurethral thulium laser enucleation of the prostate (ThuLEP) and provide references for reducing the occurrence of postoperative complications. A retrospective analysis was conducted on 103 patients with benign prostate hyperplasia who underwent ThuLEP from September 2020 to August 2022. Among them, 95 patients had complete clinical and follow-up data. Clinical data and follow-up information of the patients were collected. Univariate and multivariate logistic regression analyses were performed to identify the influencing factors of postoperative complications following ThuLEP. The incidence of postoperative complications following ThuLEP was 11.58% (11/95), among which the rates of Grade I, II, and III complications were 3.16% (3/95), 3.16% (3/95), and 5.29% (5/95), respectively. The prostate volume after ThuLEP was 96.11 ± 43.38 ml in the uncomplication group and 139.71 ± 96.41 ml in the complication group, and there was a statistically significant difference between the 2 groups (*P* = .02). The prostate mass after ThuLEP was 46.99 ± 22.14 ml in the uncomplication group and 70.25 ± 41.37 ml in the complication group, and there was a statistically significant difference between the 2 groups (*P* = .01). The postoperative catheter removal time after ThuLEP was 3 ± 1.04 days in the uncomplication group and 2 ± 1.49 days in the complication group, and there was a statistically significant difference between the 2 groups (*P* = .02).Univariate and multivariate logistic regression analyses revealed that the postoperative catheter removal time (*P* = .046) was an independent risk factor for Grade II-III complications after ThuLEP. Prostate volume and prostate mass are influencing factors for postoperative complications following ThuLEP. Postoperative catheter removal time is an independent risk factor for postoperative complications following ThuLEP.

## 1. Introduction

is a common cause of lower urinary tract symptoms (LUTS) in men, significantly impacting their quality of life.^[1,2]^ The prevalence of LUTS due to BPH ranges from 10% to 30% in men aged 60–70 years, increasing to 30% in men aged 80 years.^[3,4]^ While observation and medication are suitable for mild to moderate cases, surgical intervention is necessary for severe cases.

Transurethral resection of the prostate (TURP) is considered the gold standard for treating BPH, although its postoperative complications warrant further attention.^[5–7]^ Transurethral thulium laser enucleation of the prostate (ThuLEP) has emerged as a novel alternative to TURP in recent years, aiming to reduce postoperative morbidity.^[8,9]^ ThuLEP yields outcomes comparable to TURP but with lower complication rates and shorter hospital stays.^[10,11]^ The continuous wave mode of holmium laser facilitates tissue resection and hemostasis intraoperatively, with minimal damage to surrounding tissues due to its shallow penetration depth.^[12,13]^

Nevertheless, postoperative complications such as bleeding, urinary retention, stress urinary incontinence, bladder neck contracture, and urethral stricture are still inevitable with ThuLEP.^[14]^ Further exploration of the risk factors contributing to these complications is essential for enhancing clinicians’ awareness of postoperative complications and providing insights for treatment adjustments.

## 2. Study population

A total of 103 patients with BPH underwent ThuLEP at the Department of Urology, Peking University First Hospital, between September 2020 and August 2022. Among them, 95 patients had complete baseline and follow-up data. Postoperative complications after ThuLEP were graded according to the Clavien-Dindo classification system. In this study, there were 3 cases of Grade I complications, 3 cases of Grade II complications, 5 cases of Grade III complications, and no cases of Grade IV-V complications.

Inclusion criteria were as follows: 1. Age 50–85 years; 2. Imaging diagnosis of BPH; 3. Moderate to severe LUTS; 4. Complete baseline and follow-up data.

Exclusion criteria were as follows: 1. Patients with postoperative pathology indicating prostate cancer; 2. Abnormal coagulation function; 3. Cardiopulmonary dysfunction.

The outcome of interest in this study was the occurrence of Grade II-III complications after ThuLEP. Twenty variables, including age, body mass index(BMI), hypertension status, diabetes status, preoperative medication treatment, preoperative surgical treatment, preoperative urinary retention, presence of nodules on ultrasound, protrusion of the middle lobe, presence of cystostomy, prostate volume, prostate mass, postvoid residual, maximum flow rate, average flow rate, preoperative t-PSA, preoperative creatinine, preoperative hemoglobin, catheter removal time, and length of hospital stay, were included for risk factor analysis. Prostate volume (cm^3^) was calculated using the formula: length (cm) × width (cm) × height (cm). Urinary retention was defined as residual urine exceeding 100 ml on ultrasound.

This study was conducted in accordance with the principles of the Helsinki Declaration (revised in 2013) and was approved by the Ethics Committee of Peking University First Hospital. Informed consent for this retrospective analysis was waived by the institutional review board.

## 3. Surgical technique

After successful anesthesia, patients were placed in lithotomy position, and routine disinfection and draping were performed. The urethra was cleansed with diluted iodine solution, and a 12° Fr 26 resectoscope (Raykeen, Shanghai, China) was inserted into the urethra to observe the degree of prostate lobe hyperplasia. The urethral mucosa was incised at the 12 o’clock position, extending from the external to the internal sphincter muscle at 2/3 of its depth, reaching the circular fibers of the bladder neck. The urethral mucosa was incised anteriorly and laterally to the 1–11 o’clock positions. Blunt dissection was performed to enter the extracapsular layer of the prostate, followed by specific treatment with holmium laser prostate enucleation. The procedure involved pushing into the lateral lobes from the 3 o’clock and 9 o’clock positions, penetrating into the bladder, and excising the prostate along the bladder neck. The enucleation was performed retrogradely, and holmium laser was used to remove hyperplastic prostate tissue deep to the capsule and up to the edge of the prostate, avoiding injury to the urethral sphincter. Hemostasis was carefully performed at each bleeding point, and residual prostate tissue in the bladder was removed. The laser scope was then removed, and the bladder was compressed in the suprapubic region to ensure unobstructed urination before releasing compression. A three-way catheter (Bard, New Jersey, USA) was left in place for continuous bladder irrigation, and the procedure was completed.

## 4. Follow-up

Postoperative complications were confirmed by querying the hospital medical record system and conducting telephone follow-ups with patients to identify any complications occurring within 3 months postoperatively. The outcome of interest was the occurrence of Grade II-III complications after ThuLEP, investigating the risk factors for postoperative complications in patients with BPH undergoing ThuLEP.

## 5. Statistical analysis

Statistical analysis was performed using SPSS 22.0 (IBM Corp., Armonk, NY, USA). Continuous variables included age, BMI, prostate volume, prostate mass, postvoid residual, maximum flow rate, average flow rate, preoperative t-PSA, preoperative creatinine, preoperative hemoglobin, catheter removal time, and length of hospital stay. Qualitative variables included the presence of hypertension, diabetes mellitus, preoperative medication, preoperative surgery, preoperative urinary retention, presence of nodules under ultrasound, protrusion of the middle lobe, and preoperative cystostomy. Measures that conformed to normal distribution were expressed as mean ± standard deviation, and data with skewed distribution were described by median (extreme variance). For continuous variables, those that conformed to normal distribution were analyzed using the t test, and those that did not conform to normal distribution were analyzed using the Mann -Whitney U test. Categorical variables were analyzed using Fisher’s exact probability test. Independent risk factor analysis was performed using univariate and multivariate logistic regression analysis (*P* < .05).

## 6. Results

1. Incidence of Complications after ThuLEP

Table 1 presents the baseline characteristics of the patients. In this study there were 54 patients without ThuLEP postoperative complications and 11 patients with complications, giving a complication rate of 11.58% (11/95). Specifically, the incidence rates of Grade I complications were 3.16% (3/95). Grade II complications occurred in 3.16% (3/95) of patients. Grade III complications occurred in 5.29% (5/95) of patients.

**Table 1:** Basic characteristics of the patients.

Variable	Mean (SD) or n/N
Patients	95
Mean age (years)	69.06 ± 7.82
BMI (kg/m2)	23.66 ± 2.46
Hypertension, n (%)	
Yes	46 (48.42%)
No	49 (51.58%)
Diabetes mellitus, n (%)	
Yes	21 (22.11%)
No	74 (77.89%)
Preoperative medication, n (%)	
Yes	76 (80.00%)
No	19 (20.00%)
Urinary retention, n (%)	
Yes	57 (60.00%)
No	38 (40.00%)
Upper urinary tract fluid, n (%)	
Yes	11 (11.58%)
No	84 (88.42%)
Ultrasound nodule, n (%)	
Yes	15 (15.79%)
No	80 (84.21%)
Middle lobe prominence, n (%)	
Yes	67 (70.53%)
No	28 (29.47%)
Preoperative cystostomy, n (%)	
Yes	2 (2.11%)
No	93 (97.89%)
Prostate volume (mL)	99.78 ± 50.70
Prostate mass (g)	48.68 ± 25.15
Postvoid residual (mL)	118.33 ± 168.82
Maximum flow rate (ml/s)	8.92 ± 5.06
Average flow rate (ml/s)	3.49 ± 1.90
Preoperative t-PSA (ng/mL)	6.93 ± 6.78
Preoperative hemoglobin (g/L)	145.10 ± 12.28
Preoperative creatinine (μmol/L)	84.89 ± 15.26
Urinary catheter removal time, (days)	2.92 ± 1.13
Days of hospitalization, (days)	4.02 ± 0.63
Clavien-Dindo Grade, n (%)	
Grade I	3 (3.16%)
Grade II	3 (3.16%)
Grade III	5 (5.26%)
Grade IV-V	0 (0.00%)

BMI = body mass index, PSA = prostate specific antigen.

1. Risk Factors Analysis for Grade II-III Complications after ThuLEP

The clinical data of patients in the ThuLEP postoperative uncomplication group (n = 87) and postoperative grade II-III complication group (n = 8) are shown in Table 2. The prostate volume after ThuLEP was 96.11 ± 43.38 ml in the uncomplication group and 139.71 ± 96.41 ml in the complication group, and there was a statistically significant difference between the 2 groups (*P* = .02). The prostate mass after ThuLEP was 46.99 ± 22.14 ml in the uncomplication group and 70.25 ± 41.37 ml in the complication group, and there was a statistically significant difference between the 2 groups (*P* = .01). The postoperative catheter removal time after ThuLEP was 3 ± 1.04 days in the uncomplication group and 2 ± 1.49 days in the complication group, and there was a statistically significant difference between the 2 groups (*P* = .02). Both univariate and multivariate logistic regression analyses indicated that postoperative catheter removal time (*P* = .046) was an independent risk factor for Grade II-III complications post-ThuLEP (Table 3).

**Table 2:** Comparison of complicated and uncomplicated group after ThuLEP.

Variable	Complicated group	Uncomplicated group	*p* value
Patients, n (%)	8 (8.42%)	87 (91.58%)	
Mean age (years)	72.63 ± 6.88	68.74 ± 7.78	0.184
BMI (kg/m2)	23.80 ± 2.50	23.65 ± 2.44	0.871
Hypertension, n (%)			0.716
Yes	3 (37.50%)	43 (49.43%)	
No	5 (62.50%)	44 (50.57%)	
Diabetes mellitus, n (%)			1
Yes	2 (25.00%)	19 (21.84%)	
No	6 (75.00%)	68 (78.16%)	
Preoperative medication, n (%)			0.216
Yes	6 (75.00%)	70 (80.46%)	
No	2 (25.00%)	17 (19.54%)	
Urinary retention, n (%)			0.709
Yes	4 (50.00%)	53 (60.92%)	
No	4 (50.00%)	34 (39.08%)	
Upper urinary tract fluid, n (%)			1
Yes	1 (12.50%)	10 (11.49%)	
No	7 (87.50%)	77 (88.51%)	
Ultrasound nodule, n (%)			0.11
Yes	3 (37.50%)	12 (13.79%)	
No	5 (62.50%)	75 (86.21%)	
Middle lobe prominence, n (%)			0.229
Yes	4 (50.00%)	63 (72.41%)	
No	4 (50.00%)	24 (27.59%)	
Preoperative cystostomy, n (%)			0.162
Yes	1 (12.50%)	1 (1.15%)	
No	7 (87.50%)	86 (98.85%)	
Prostate volume (mL)	139.71 ± 96.41	96.11 ± 43.38	0.02
Prostate mass (g)	70.25 ± 41.37	46.99 ± 22.14	0.01
Postvoid residual (mL)	58.83 ± 31.76	124.65 ± 175.83	0.37
Maximum flow rate (ml/s)	8.7 ± 2.92	8.95 ± 5.27	0.91
Average flow rate (ml/s)	3.4 ± 1.58	3.51 ± 1.93	0.89
Preoperative t-PSA (ng/mL)	4.46 ± 2.86	7.17 ± 6.99	0.29
Preoperative hemoglobin (g/L)	141.38 ± 16.38	145.44 ± 11.64	0.38
Preoperative creatinine (μmol/L)	81.89 ± 9.89	85.16 ± 15.60	0.57
Urinary catheter removal time, (days)	2 ± 1.49	3 ± 1.04	0.02
Days of hospitalization, (days)	4 ± 0.82	4.02 ± 0.60	0.92

**Table 3:** Univariate and multivariate logistic regression analysis for complications occur.

	Univariate		Multivariate	
Variable	OR (95%CI)	*p* value	OR (95%CI)	*p* value
Mean age	1.604 (0.970–1.167)	0.188		
BMI	1.025 (0.763–1.378)	0.869		
Hypertension	1.629 (0.366–7.240)	0.522		
Diabetes mellitus	0.838 (0.156–4.494)	0.837		
Preoperative medication	1.373 (0.254–7.407)	0.713		
Urinary retention	0.935 (0.210–4.170)	0.93		
Upper urinary tract fluid	0.909 (0.101–8.175)	0.932		
Ultrasound nodule	0.267 (0.056–1.264)	0.096		
Middle lobe prominence	2.625 (0.608–11.343)	0.196		
Preoperative cystostomy	0.081 (0.005–1.445)	0.087		
Prostate volume	1.012 (1.000–1.023)	0.045	1.008 (0.977–1.039)	0.626
Prostate mass	1.024 (1.000–1.048)	0.05	1.008 (0.946–1.075)	0.797
Postvoid residual	0.996 (0.988–1.005)	0.393		
Maximum flow rate	0.990 (0.846–1.159)	0.905		
Average flow rate	0.971 (0.638–1.476)	0.889		
Preoperative t-PSA	0.905 (0.752–1.089)	0.289		
Preoperative hemoglobin	0.975 (0.921–1.031)	0.375		
Preoperative creatinine	0.983 (0.928–1.042)	0.563		
Urinary catheter removal time	0.578 (0.355–0.943)	0.028	0.582 (0.343–0.989)	0.046
Days of hospitalization	0.944 (0.297–3.002)	0.922		

## 7. Discussion

In the past few decades, TURP has been the gold standard surgical treatment for benign prostatic hyperplasia (BPH) with prostate volumes ranging from 30 to 80 ml, while open prostatectomy has been reserved for prostates larger than 80 ml.^[15,16]^ However, with the evolution of laser therapies, ThuLEP has emerged as the gold standard treatment for large prostates (>80 mL) and an alternative for medium-sized prostates (30–80 mL).^[17]^ This study aimed to explore the risk factors associated with postoperative complications following ThuLEP. A better understanding of these potential complications will assist urologists in providing appropriate counseling to patients and identifying and managing these conditions effectively.

Common postoperative complications following prostate surgery include fever, bleeding, electrolyte imbalances, urinary incontinence, and urethral stricture.^[8,18]^ With the diversification of surgical techniques for BPH, several studies have investigated the occurrence of postoperative complications associated with different surgical approaches. Palmisano et al^[19]^ conducted a single-center series study involving 160 patients undergoing TURP for BPH, with postoperative hospital readmission primarily due to hematuria (6.8%) and infection (4.3%). A meta-analysis by Friedrich O Hartung et al^[11]^ found a superiority of holmium laser over thulium laser in terms of postoperative urinary incontinence (*P* = .05). In a study by Bach et al,^[20]^ it was found that compared to Holmium laser enucleation of the prostate (HoLEP) , ThuLEP was associated with lower rates of postoperative blood transfusion, urinary retention, and urinary incontinence, possibly due to the deeper penetration of holmium laser compared to thulium laser and tissue tearing caused by Ho:YAG pulse emission. In our study, Grade I complications were all postoperative fever, with an incidence rate of 3.16% (3/95), which is consistent with previous studies.

Although complications such as postoperative fever and electrolyte imbalances may cause significant distress to patients, they do not pose significant physical harm. However, Grade II-III complications, such as blood transfusion post-bleeding and repeat surgery for stricture, impose substantial financial burdens on patients and significant physical strain. In this study, we analyzed risk factors for Grade II-III complications post-ThuLEP, which included postoperative blood transfusion and repeat surgery for stricture. The incidence rates of Grade II and III complications were 3.16% (3/95) and 5.29% (5/95), respectively. Our analysis revealed that postoperative catheter removal time (*P* = .046) was an independent risk factor for Grade II-III complications post-ThuLEP, while prostate volume (*P* = .02) and prostate mass (*P* = .01) were influencing factors.

Castellani et al^[21]^ analyzed 1303 patients undergoing ThuLEP for BPH and found that the incidence rate of postoperative urethral stricture was 4.98% (50/1303), with prostate volume identified as a risk factor for postoperative urethral stricture. Similarly, Anton Grechenkov et al^[22]^ found that prostate size was a risk factor for postoperative urethral stricture in patients undergoing surgery for BPH. Our study is consistent with previous research trends, as we also found that prostate volume and mass are associated with the occurrence of complications post-ThuLEP. This may be attributed to the increased surgical difficulty associated with larger prostates, which may require more extensive resection or manipulation to complete the procedure. This increases the risk of urethral injury and bleeding during the surgery. Surgeries on larger prostates may be associated with infections or other complications involving surrounding tissues, which may increase the risk of urethral stricture.

Agarwal et al^[23]^ demonstrated that early catheter removal after HoLEP for BPH is a viable option, but our study found that early catheter removal may lead to postoperative complications. The presence of the catheter may cause irritation and damage to the urethra, especially when the catheter is removed early. Insufficient time for the urethra to recover may increase the risk of urethral stricture. Shorter catheterization time may affect the recovery process of the urethra postoperatively. Early catheter removal may result in inadequate restoration of the normal structure and function of the urethra, thereby increasing the risk of urethral stricture.

Our study has certain limitations. It is a retrospective study, with some missing data on factors influencing postoperative complications. Additionally, it is a single-center study, which may introduce certain biases.

## 8. Conclusions

Prostate volume and mass are influencing factors for the occurrence of complications post-ThuLEP. Postoperative catheter removal time is an independent risk factor for Grade II-III complications post-ThuLEP.

## Author contributions

**Methodology:** Chao Zuo, Wenzhi Gao.

**Project administration:** Chao Zuo.

**Software:** Chao Zuo.

**Writing – original draft:** Chao Zuo, Wenzhi Gao, Kai Zhang.

**Writing – review & editing:** Chao Zuo, Kai Zhang.

**Investigation:** Wenzhi Gao, Minghua Zhang.

**Formal analysis:** Yisen Meng, Xuebing Meng.

**Data curation:** Kunlin Yang.

**Resources:** Kai Zhang.

**Supervision:** Kai Zhang.
